# U.K. standards of care for occupational contact dermatitis and occupational contact urticaria

**DOI:** 10.1111/bjd.12256

**Published:** 2013-06-05

**Authors:** A Adisesh, E Robinson, PJ Nicholson, D Sen, M Wilkinson, on behalf of the Standards of Care Working Group

**Affiliations:** 1 Centre for Workplace Health, Health & Safety LaboratoryHarpur Hill, Buxton, SK17 9JN, U.K; 2 Procter & GambleRusham Park, Whitehall Ln, Egham, Surrey, TW20 9NW, U.K; 3 Health & Safety ExecutiveRedgrave Court, Merton Road, Bootle, Merseyside, L20 7HS, U.K; 4 Leeds General InfirmaryGreat Geore St, Leeds, West Yorkshire, LSI 3EX, U.K

## Abstract

The diagnosis of occupational contact dermatitis (OCD) and occupational contact urticaria (OCU) is a process that involves fastidious clinical and occupational history taking, clinical examination, patch testing and skin-prick testing. A temporal relationship of work and/or the presence of a rash on the hands only raises suspicion of an occupational cause, and does not necessarily confirm an occupational causation. The identification of allergy by patch or prick tests is a major objective, as exclusion of an offending allergen from the environment can contribute to clinical recovery in the individual worker and avoidance of new cases of disease. This can be a complex process where allergens and irritants, and therefore allergic and irritant contact dermatitis, may coexist. This article provides guidance to healthcare professionals dealing with workers exposed to agents that potentially cause OCD and OCU. Specifically it aims to summarize the 2010 British Occupational Health Research Foundation (BOHRF) systematic review, and also to help practitioners translate the BOHRF guideline into clinical practice. As such, it aims to be of value to physicians and nurses based in primary and secondary care, as well as occupational health and public health clinicians. It is hoped that it will also be of value to employers, interested workers and those with responsibility for workplace standards, such as health and safety representatives. Note that it is not intended, nor should it be taken to imply, that these standards of care override existing statutory and legal obligations. Duties under the U.K. Health and Safety at Work Act 1974, the Management of Health and Safety at Work Regulations 1999, the Control of Substances Hazardous to Health Regulations 2002, the Equality Act 2010 and other relevant legislation and guidance must be given due consideration, as should laws relevant to other countries.

Skin disease represents 10–40% of recognized occupational diseases in the European Union.^1^ Contact dermatitis accounts for 70–90% of all occupational skin disease, and contact urticaria for < 10%. Other occupational skin disorders include folliculitis/acne, infections, neoplasia, hyperpigmentation and vitiligo. Up to half of workers with occupational contact dermatitis (OCD) experience adverse effects on quality of life (QoL), daily function and relationships at home.^2^

Occupational allergic disease in the U.K. was the focus of a House of Lords Science and Technology Committee inquiry.^3^ A standard of care was recommended to improve the education and knowledge of medical practitioners in the diagnosis and treatment of these diseases. In response, a systematic, evidence-based review for OCD and occupational contact urticaria (OCU) was published by the British Occupational Health Research Foundation (BOHRF)^2^ (Table 1). These standards of care were produced to help practitioners translate the BOHRF guideline into clinical practice.

**Table 1:** Key recommendations from the British Occupational Health Research Foundation (BOHRF) Systematic Review: Occupational Contact Dermatitis and Urticaria

Employers and their health and safety personnel should:	
1	Implement programmes to remove or reduce exposure to agents that cause OCD and OCU
2	Provide appropriate gloves and cotton liners where the risk of developing OCD or OCU cannot be eliminated by removing exposure to its causes
3	Make after-work (conditioning) creams readily available in the workplace and encourage workers to use them regularly
4	Do not promote the use of prework (barrier) creams, as this may confer on workers a false sense of security and encourage them to be complacent in following more effective preventative measures
5	Provide workers with appropriate health and safety information and training
6	Ensure that workers who develop OCD or OCU are properly assessed by a physician who has expertise in occupational skin disease for recommendations regarding appropriate workplace adjustments
Health practitioners should:	
7	Ask a worker who has been offered a job that will expose them to causes of OCD if they have a personal history of dermatitis, particularly in adulthood, and advise them of their increased risk, and care for and protect their skin
8	Ask the worker who has been offered a job that will expose them to causes of OCU if they have a personal history of atopy and advise them of their increased risk, and care for and protect their skin
9	Take a full occupational history whenever someone of working age presents with a skin rash, asking about their job, the materials with which they work, the location of the rash and any temporal relationship with work
10	Arrange for a diagnosis of OCD or OCU to be confirmed objectively (patch tests and/or skin-prick tests) and not on the basis of a compatible history alone because of the implications for future employment

OCD, occupational contact dermatitis; OCU, occupational contact urticaria. Each recommendation was formed from a number of evidence-based statements, graded using both the Scottish Intercollegiate Guidance Network system and the Royal College of General Practitioners' 3-star system (modified in 2008 by the Swedish Council on Technology Assessment in Health Care report for scientific studies). Full details of the rating systems used and the grades of evidence applied to each statement are available in the BOHRF review.^2^

While OCD and OCU can be prevented by applying a hierarchy of occupational control measures [i.e. hazard elimination, substitution, engineering controls, safe work practices and, where this is not possible, personal protective equipment (PPE)], the problem remains significant, affecting workers across a range of industries and activities. It is important to distinguish between occupational skin disease (caused directly or made worse by work) and nonoccupational skin disease, as well as allergic and irritant causes, as the treatment and occupational management will differ.

The prognosis of OCD varies widely, but reasonable control of symptoms and job retention are possible in some occupational settings. Similar proportions of patients report either improvement or complete resolution, or ongoing symptoms. After removal from exposure, a small proportion of patients have persistent post-occupational dermatitis. A loss of job or complete change of employment is common among workers with OCD; however, most manage to continue working in some capacity.^2^ There is little evidence related to the prognosis of OCU.

## Scope and background

Guidance from the BOHRF review was aimed at managers, workers and health and safety professionals. This evidence base was used to develop a standard of care document for OCD and OCU, following review and discussion by the Standards of Care Working Group formed for this purpose (details of this group and their affiliations are provided in the acknowledgments section). The information and guidance offered in this document is restricted to OCD and OCU only. The terms OCD and OCU here encompass those situations where a pre-existing condition or tendency is worsened by exposures at work. Other recent reviews have been completed by National Health Service (NHS) Plus and the Royal College of Physicians of London for latex allergy and dermatitis,^4^,^5^ and guidelines for the management of contact dermatitis^6^ were commissioned by the British Association of Dermatologists.

Clinicians, employers and workers need to exercise judgement, knowledge and expertise when deciding whether to apply any guidelines, taking into account individual circumstances and patients' wishes. Clinical judgement is necessary when using evidence statements to guide decision making. Limited recommendations on a particular issue or effect do not necessarily mean that it is untrue or unimportant, but may simply reflect insufficient evidence.

## Prevention

### Primary prevention

Primary prevention aims to avert the onset of disease. The most effective primary prevention measure is substitution with a less harmful material, followed by engineering and hygiene measures supported by a comprehensive risk assessment. Reducing allergen exposure, such as by substituting latex gloves for powder-free, low-protein alternatives,^4^ or by adding ferrous sulfate to cement,^7^,^8^ has decreased the incidence of OCU and OCD.

### Secondary prevention

Secondary prevention aims to detect disease at an early or presymptomatic stage, for example by health surveillance. If early indications of skin conditions are identified, removal from exposure may lead to regression of these symptoms and prevent progression to established disease. However, this approach should be supported by a clear occupational health protocol that defines a line of referral or investigation for those workers with skin problems. Where there is a suspicion of disease, it is likely that further health assessment may be required to confirm a diagnosis, although this may depend upon local expertise.

### Tertiary prevention

Tertiary prevention aims to mitigate and treat the effects of established disease, being largely concerned with treatment and reducing disability in workers already diagnosed with a condition. The standard advice given is that further exposure to known causative agents is reduced or eliminated. Continued exposure may be allowed depending on the particular agent and the severity of the skin condition, but with improved controls and therefore better compliance with legal requirements, for example the Control of Substances Hazardous to Health (COSHH) Regulations 2002 (as amended).^9^ However, workers must understand the potential consequences of this approach, exposure must be controlled to the lowest possible levels and the worker must be enrolled into a health-surveillance programme. In many cases, medical and occupational management will occur at the primary care level, at least initially.

## General care

Other measures include the use of skin-conditioning creams or emollients and prework creams (often referred to as barrier creams). The term ‘barrier cream’ may unfortunately encourage its use as a substitute for more proven preventative approaches (such as gloves or exposure control). Evidence of barrier creams' effectiveness at preventing irritant contact dermatitis is mixed, with certain creams offering only limited protection against certain specific exposures.^10^–^12^ Emollients are lotions, creams or ointments intended to moisturize the skin and replace lipids, to help maintain the functional integrity of healthy skin. The optimum timing and frequency of emollient application is unclear, although studies have demonstrated beneficial effects from their regular use.^13^–^16^ However, there is some evidence that use of skin-conditioning creams helps to prevent the development of OCD.^13^–^16^

**Standard**

There should be no use of prework creams labelled or promoted as ‘barrier creams’.

**Standard**

Skin-conditioning creams should be available at hand-washing areas and in other appropriate places. Training and guidance in the application of skin-conditioning creams should be provided.

### Management of the problem

Diagnosing OCD and OCU is a process requiring time and access to key resources. Care must be taken to distinguish occupational disease from non-OCD and endogenous eczema, and irritant from allergic OCD, as the occupational management of the patient will differ. The tests used for investigating allergic response to occupational exposure within dermatology clinics include patch tests, skin-prick tests and specific IgE measurement.^17^,^18^ Practitioners should refer to appropriate guidelines for details of these tests.^18^

#### The patient journey

**Fig. 1:**
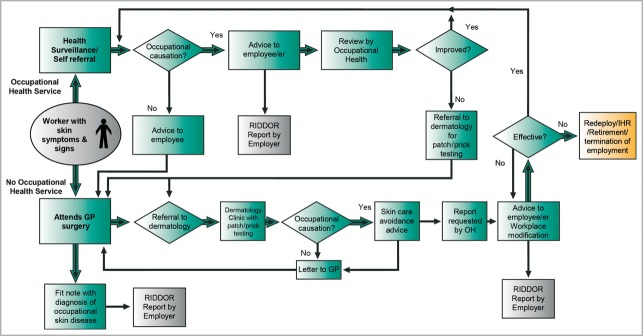
Patient journey schematic for workers with occupational skin disease (occupational contact dermatitis and occupational contact urticaria). GP, general practitioner; IHR, ill-health retirement; RIDDOR, Reporting of Injuries, Diseases and Dangerous Occurrences Regulations 1995; OH, occupational health.

The typical patient journey moves from a period of exposure while asymptomatic to a final diagnosis. Figure 1 plots the journey for workers experiencing skin signs and symptoms, and the potential routes dependent on their enrolment in a workplace health surveillance scheme. Workers with an occupational health service (OHS) may present directly by self-referral if a skin rash or symptoms start in between scheduled health surveillance. In the absence of an OHS, the patient journey takes a path starting with an initial visit to their general practitioner (GP). Education of employees in the recognition of dermatitis and urticaria is important to encourage early presentation. Equally, it is crucial that primary care services are alerted to the potential for occupational causation or exacerbation of skin disease.

An OHS should be familiar with the work activity, processes and substances that an employee uses, to advise any changes to benefit skin care. When this advice has not resolved the problem within a reasonable period of time, e.g. 3 months, referral for further investigation by patch and prick testing is essential to exclude unsuspected contact allergy. An OHS, with the employee's consent, will often need to liaise with the GP to facilitate referral. When a diagnosis of OCD or OCU is made by a doctor, and the employer receives a diagnosis in writing, the employer is obliged to make a report of a case of disease to the Health and Safety Executive (HSE), where the circumstances as outlined in the Reporting of Injuries, Diseases and Dangerous Occurrences Regulations 1995 (RIDDOR)^19^ apply (Table 2). As reporting to the HSE, or the relevant enforcing authority, can occur only when a doctor has seen an employee, OHSs need robust processes for access to competent medical advice. The OHS may also need to liaise with secondary care to obtain a dermatologist's report with the employee's consent. An OHS can provide useful assistance to the dermatologist in identifying the circumstances of the work and the workplace substances that an employee may be using, for example by providing Material Safety Data Sheets or more detailed information. Once a diagnosis of allergic contact dermatitis (ACD) or allergic contact urticaria (ACU) has been concluded, the OHS can also help with ensuring that sources of allergen exposure are recognized, removed or reduced. For irritant exposures, the OHS can help with advice on avoidance or reduction. The OHS should also follow up the affected employee either to ensure resolution of the problem or to support further job modification or redeployment.

**Table 2:** Reporting requirements for occupational dermatitis under Reporting of Injuries, Diseases and Dangerous Occurrences Regulations 1995 (RIDDOR)

HSE32. RIDDOR – Information for doctors	
Reportable diseases from Schedule 3 of the regulations	
Occupational diseases – conditions due to substances	
Section 45: occupational dermatitis	
Activity: work involving exposure to any of the following agents	
a	Epoxy resin systems
b	Formaldehyde and its resins
c	Metalworking fluids
d	Chromate (hexavalent and derived from trivalent chromium)
e	Cement, plaster or concrete
f	Acrylates and methacrylates
g	Colophony (rosin) and its modified products
h	Glutaraldehyde
i	Mercaptobenzothiazole, thiurams, substituted paraphenylenediamines and related rubber-processing chemicals
j	Biocides, antibacterials, preservatives or disinfectants
k	Organic solvents
l	Antibiotics and other pharmaceuticals and therapeutic agents
m	Strong acids, strong alkalis, strong solutions (e.g. brine) and oxidizing agents including domestic bleach or reducing agents
n	Hairdressing products including in particular dyes, shampoos, bleaches and permanent waving solutions
o	Soaps and detergents
p	Plants and plant-derived material including in particular the daffodil, tulip and chrysanthemum families, the parsley family (carrots, parsnips, parsley and celery), garlic and onion, hardwoods and the pine family
q	Fish, shellfish or meat
r	Sugar or flour
s	Any other known irritant or sensitizing agent including in particular any chemical bearing the warning ‘may cause sensitization by skin contact’ or ‘irritating to the skin’

Note that urticaria is not listed in the RIDDOR guidance.

The aim should be for the employee to remain in work without needing continued medical treatment. In some circumstances there may be few options for avoidance of an allergen or for redeployment, particularly if the employer is a small enterprise. As a last resort, the possibility of retirement due to ill health, or termination of employment by the employer may arise. Feedback to the workplace to effect appropriate supportive changes should occur following a diagnosis. Employers have a duty to report diagnoses of occupational skin disease to the HSE under RIDDOR. In practice, this may not always happen. However, when an employer receives a fit note (MED3) stating work-related dermatitis or eczema as the diagnosis, this would require reporting by the employer (Table 2). While employers should be aware of any cases that have been diagnosed in their workforce, this information may not always be disclosed by healthcare professionals, particularly if the worker has not given consent.

All patients must be asked about work in relation to their skin symptoms to avoid missed diagnosis of OCD and OCU. A decision must be made locally as to the extent of any investigation prior to possible referral for specialist advice. In practice, medical history and physical examination may be carried out in nonspecialist centres and by a GP, with skin-prick or patch testing following referral to a specialist centre. An occupational health professional with knowledge of an employee's workplace may be in a position to effect change that resolves many early cases of OCD by ensuring good skin-care practices. The involvement of a dermatologist will usually be needed to confirm or exclude ACD and ACU, as well as for more persistent cases.

**Standard**

Arrangements for access to a physician who has expertise in occupational skin disease should be in place for initial diagnosis and recommendations regarding appropriate workplace adjustments, together with subsequent investigation by patch or prick testing if appropriate.

#### Diagnostic management

Confirming diagnoses of OCD and OCU requires skill in taking a medical and occupational history. While the hands, and thereafter the arms and face, are most commonly affected by OCD and OCU, such distributions are not confined to occupational disease and only help to inform the diagnosis. Similarly, symptoms improving away from work can produce false-positive diagnoses, so further validation is needed.

**Standard**

Whenever someone of working age presents with a skin rash the clinical records should contain a full clinical and occupational history asking about their job, the materials with which they work, the location of the rash and any temporal relationship with work.

The work-relatedness of symptoms and signs is the best guide to whether contact dermatitis or contact urticaria is aggravated or caused by work.^20^ However, pre-existing dermatitis (atopic eczema) can be aggravated particularly by irritant exposures at work.^20^ Occupational dermatitis can also develop a chronic state where the work-relatedness becomes less obvious.

Observation of the rash before exposure or treatments modify the appearance aids diagnosis. Other techniques often used include the following. (i) Skin-prick or blood tests for specific IgE, which are available for investigating contact urticaria for most high- and some low-molecular-weight allergens. Few standardized allergens are commercially available, limiting their use. (ii) Patch-test agents for a wide range of standardized contact allergens (usually of low-molecular-weight) are available commercially. However, a positive test merely denotes sensitization, which can occur with or without disease. When patch tests to relevant potential allergens are negative, contact dermatitis is then diagnosed as irritant in origin.

**Standard**

The diagnosis of suspected occupational skin disease (contact dermatitis or contact urticaria) should include objective patch or prick testing where (i) the condition has not improved 3 months after initial advice; and (ii) a contact allergy is suspected or there are implications for fitness to work, such as altered employment, loss of job or complete change of employment.

### Health surveillance and education

There is no direct evidence derived from studies in working populations about the effectiveness of health surveillance in the early detection of OCD or OCU, or comparing different screening methods.^5^

**Fig. 2:**
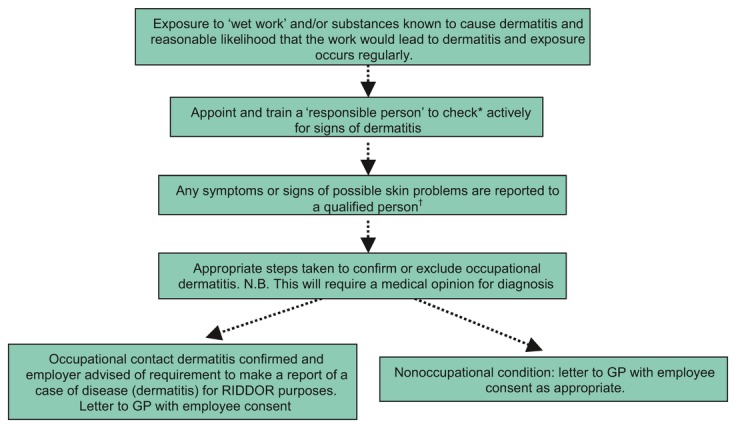
Health surveillance for occupational dermatitis. GP, general practitioner; RIDDOR, Reporting of Injuries, Diseases and Dangerous Occurrences Regulations 1995. *This may be performed by direct inspection and/or by questionnaire. ^†^A qualified person is a suitably qualified medical practitioner or occupational health nurse.

A health surveillance programme (Fig. 2) would normally be expected in a workplace where a risk assessment (a legal requirement in Great Britain under COSHH regulations) has identified a significant risk from skin sensitizers or irritants.

Recognition of OCD and OCU in workers at risk of these conditions is a shared responsibility of all professionals in contact with them, in addition to any workplace health surveillance. Several studies have demonstrated the benefits of education in the reduction of occupational dermatitis;^21^–^23^ appropriately targeted and sustained educational interventions can induce important behavioural changes.^4^ In addition, workers should be informed about how workplace agents can cause adverse skin conditions and how to reduce exposure to causative agents. It should also be made clear to workers what action to take should they develop symptoms, particularly if these occur between scheduled health surveillance visits.

**Standard**

Where a worker has been offered a job that will expose them to causes of OCD, the clinical records should indicate if they have a personal history of dermatitis, particularly in adulthood, and record advice given to them of their increased risk, and how to care for and protect their skin.

**Standard**

Where a worker has been offered a job that will expose them to causes of OCU, the clinical records should indicate if they have a personal history of atopy, and record advice given to them of their increased risk, and how to care for and protect their skin.

Any educational programmes should be aimed not only at employers and workers but also at a variety of healthcare professionals including nurses and doctors (based in industry and primary and secondary care), occupational hygienists and workers with responsibility for health and safety. The programme for workers should include an element of continuing education, although not necessarily delivered at the same time as the skin health surveillance. The HSE website on occupational skin disease (http://www.hse.gov.uk/skin/index.htm) is an excellent resource for employees, employers and health practitioners.

## Workplace intervention

### Exposure control

Risk assessment in the workplace is designed to identify all potential hazards, for example ‘wet work’, and assess the associated risk, thereby minimizing workplace risk. This process normally occurs as part of a COSHH assessment; further details are available on the HSE website or at COSHH essentials (http://www.coshh-essentials.org.uk). While hygiene is beyond the scope of this article, the principles of occupational hygiene should be adhered to as discussed under ‘Primary prevention’.

Once a worker has developed OCD or OCU, the question remains regarding the most appropriate occupational management. Most studies concentrate on primary prevention as it is the most effective measure, rather than investigating actions to allow an individual to continue working with the causative agent. Although several interventions have been evaluated, the changes to work practices that were most effective, such as the careful and rapid changing of contaminated clothing^24^ or the use of disposable towels rather than contaminated rags,^25^ are not often mentioned.

Evidence shows that continued exposure to the causative agent causes ongoing symptoms.^26^ Avoidance of relevant irritants and allergens can be effective in improving or resolving the dermatitis.^27^–^29^ However, persistent dermatitis may occur despite adequate avoidance of allergens or irritants, even after several years. This phenomenon is particularly marked for ACD to chromate and other metal salts.^30^,^31^ In many workplaces both irritants and allergens are present. Therefore, the specific allergen or irritant must be considered when advising on avoidance of exposure and recovery. For instance, the NHS Plus review of latex allergy^4^ reported three studies showing that changing from powdered to powder-free latex gloves was associated with significant reduction in symptoms, disease-severity indicators and immunological markers of sensitization. PPE has a role where other controls leave a significant residual risk, or are not feasible. Studies of preventive measures are complicated because they are usually implemented as a broad programme with many components, and distinguishing the relative effect of one measure against another is difficult.

**Standard**

Employers have legal duties to assess the health risks from skin exposure to hazardous substances at work. They should prevent or, where this is not reasonably practicable, adequately control exposure to the hazards by using and maintaining suitable controls.

### Workplace modification and redeployment

Modifying the workplace or work tasks can reduce exposure; however, the evidence is limited. In one large study and one small case series of OCD, advice about work practices, PPE or job changes appeared to make no difference to clinical improvement.^32^,^33^ Conversely, six small case series in specific occupational settings found a positive outcome in workers with OCD or OCU from redeployment,^34^ introduction of exposure controls,^35^,^36^ or use of protective clothing or gloves.^25^,^27^,^35^ Glove use reduces dermatitis, enabling workers to continue in the same occupation.^24^–^27^,^29^ However, sometimes gloves can worsen irritant hand dermatitis, although using cotton-lined gloves may mitigate the effects by preventing the reduction of skin-barrier function attributed to long-term occlusive glove use.^14^

Personal protective equipment offers protection only when selected and stored correctly, worn properly, removed safely and either replaced or maintained regularly. It should be noted that some PPE, including powdered, high-protein latex gloves, can cause OCD and OCU or exacerbate existing conditions.^4^ Limiting gloves to use only where necessary helps to prevent these additional complications. The correct selection of PPE includes ensuring that the type of gloves is appropriate to the circumstances of use, for example to avoid chemical permeation or to have a sufficient cuff length.

**Standard**

Where adequate control of exposure cannot be achieved by other means, suitable PPE should be provided in combination with other measures. The use of gloves must take into account appropriate selection and training on glove usage, including the provision of cotton liners.

### Worker education

The outcome of different educational intervention programmes varies, with some having no demonstrable effect.^32^ Individuals demonstrating knowledge of their diagnosis and its causes have less dermatitis and are more likely to show improvement than those who are not informed,^37^ and nurse-led education for irritant OCD has shown better outcomes.^38^

**Standard**

Information and training aimed at improving and maintaining skin health should be provided to employees at risk of developing OCD or OCU at the time of employment and regularly thereafter.

### Psychosocial considerations

Workplaces have complex social and psychological dynamics that interventions must take into account. Barriers to early recognition and reporting of OCD include failure to acknowledge work causation, workers' fears for continued employment, and a perception that ‘it won't happen to me’. Peer pressure from coworkers (both positive and negative) is likely to be important in determining behaviours and compliance with workplace regulations. As a consequence, it is essential to involve all staff, unions and health and safety advisors in any decisions.

Agner *et al*.^39^ reported a significant correlation between increased severity of hand eczema and reduced QoL. QoL was reduced in all 10 studies reviewed for the effect of contact dermatitis on QoL.^40^ The same authors noted that ‘hand involvement has a considerable impact on QoL’, and ‘an early, confirmed diagnosis is associated with improved QoL’; reduced QoL due to contact dermatitis also predicted psychiatric comorbidity such as depression and anxiety.

From the employer's point of view, some have concerns about the costs associated with a case of occupational disease, including absenteeism, potential compensation claims and increased liability insurance. In such cases, there may be less incentive for reporting, and compensation systems may lead to reduced reporting rates for OCD and OCU.

## Medicolegal aspects

Dermatitis is a medical condition listed as a Prescribed Disease (D5) for social security purposes. Eligible claimants receive Industrial Injuries Disablement Benefit, paid to people who become disabled because of an accident at work, or who have certain prescribed diseases caused by their job. The patient should be advised to contact their local social security office for further information. The payment depends on the degree of disability and may be additive to any other existing Prescribed Disease.

The Equality Act 2010 protects anyone who has, or has had, a disability. Direct discrimination occurs where, because of disability, a person receives worse treatment than someone who does not have a disability. This provision is intended to stop people from being denied a service, or from receiving a worse service, because of prejudice.

Civil litigation may occur when an employee pursues a legal claim for personal injury where they believe their employer was negligent. In such situations medical records can be helpful in confirming, or refuting, the cause, and may assist in identifying the likely exposures.

## Audit

Healthcare professionals and organizations should audit their practice; however, to do so agreed standards are needed. These standards of care for OCD and OCU (Table 3) may form the basis for a clinical audit, which should be encouraged within primary and secondary care as well as in OHSs. Compliance with all statutory responsibilities is required and should be documented.

**Table 3:** Summary: standards of care for occupational contact dermatitis (OCD) and occupational contact urticaria (OCU)

**Standards of care: employer**	
1	There should be no use of prework creams labelled or promoted as ‘barrier creams’
2	Skin-conditioning creams should be available at hand-washing areas and in other appropriate places. Training and guidance in the application of skin-conditioning creams should be provided
3	Arrangements for access to a physician who has expertise in occupational skin disease should be in place for initial diagnosis and recommendations regarding appropriate workplace adjustments, together with subsequent investigation by patch or prick testing if appropriate
4	Employers have legal duties to assess the health risks from skin exposure to hazardous substances at work. They should prevent or, where this is not reasonably practicable, adequately control exposure to the hazards by using and maintaining suitable controls
5	Where adequate control of exposure cannot be achieved by other means, suitable personal protective equipment should be provided in combination with other measures. The use of gloves must take into account appropriate selection and training on glove usage, including the provision of cotton liners
6	Information and training aimed at improving and maintaining skin health should be provided to employees at risk of developing OCD or OCU at the time of employment and regularly thereafter
**Standards of care: health professional**	
7	Whenever someone of working age presents with a skin rash the clinical records should contain a full clinical and occupational history asking about their job, the materials with which they work, the location of the rash and any temporal relationship with work
8	The diagnosis of suspected occupational skin disease (OCD or OCU) should include objective patch or prick testing where (i) the condition has not improved 3 months after initial advice, and (ii) a contact allergy is suspected or there are implications for fitness to work, such as altered employment, loss of job or complete change of employment
9	Where a worker has been offered a job that will expose them to causes of OCD, the clinical records should indicate if they have a personal history of dermatitis, particularly in adulthood, and record advice given to them of their increased risk, and how to care for and protect their skin
10	Where a worker has been offered a job that will expose them to causes of OCU, the clinical records should indicate if they have a personal history of atopy, and record advice given to them of their increased risk, and how to care for and protect their skin

The following details should be clearly documented in case notes: (i) a full list of occupations held and the likely associated occupational exposures; (ii) advice to patients about continuing employment once a diagnosis has been made; and (iii) compensation advice appropriate to the case.

Additionally, details should be noted in a COSHH health record, as stipulated by the U.K. COSHH regulations (http://www.hse.gov.uk/coshh/basics/surveillance.htm).

## Future developments

It will be important that any future research relevant to the diagnosis and management of OCD and OCU, causative agents, mechanisms of action and health effects be considered in addition to the information provided here. This will ensure that the current standards proposed (summarized in Table 3) are amended and updated in accordance with current best practice.

**What's already known about this topic?**

- Occupational contact dermatitis (OCD) and occupational contact urticaria (OCU) remain prevalent among U.K. workers and affect quality of life and workability.
- Despite extensive research, the prognosis for workers remains variable, with differing outcomes on health and employment.

**What does this study add?**

- These standards of care aim to improve the education and knowledge of medical practitioners in the diagnosis and management of OCD and OCU, offering a practical tool to improve the consistency and quality of diagnosis and care.

## References

[1] European Agency for Safety and Health at Work (2007). Occupational Skin Diseases and Dermal Exposure in the European Union (EU-25): Policy and Practice Overview.

[2] Nicholson PJ, Llewellyn D (2010). Occupational Contact Dermatitis and Urticaria.

[3] House of Lords Science and Technology Committee

[4] NHS Plus, Royal College of Physicians, Faculty of Occupational Medicine (2008). Latex Allergy: Occupational Aspects of Management. A National Guideline.

[5] NHS Plus, Royal College of Physicians, Faculty of Occupational Medicine (2009). Dermatitis: Occupational Aspects of Management. A National Guideline.

[6] Bourke J, Coulson I, English J (2009). Guidelines for care of contact dermatitis. Br J Dermatol.

[7] Avnstorp C (1989). Prevalence of cement eczema in Denmark before and since addition of ferrous sulphate to Danish cement. Acta Derm Venereol.

[8] Roto P, Sainio H, Reunala T, Laippala P (1996). Addition of ferrous sulphate to cement and risk of chromium dermatitis among construction workers. Contact Dermatitis.

[9] Health and Safety Executive (2001). Control of Substances Hazardous to Health (COSHH) Regulations.

[10] Bendsöe N, Björnberg A, Löwhagen GB, Tengberg JE (1987). Glass fibre irritation and protective creams. Contact Dermatitis.

[11] Elsner P (2007). Skin protection in the prevention of skin diseases. Curr Probl Dermatol.

[12] Krajewska D, Rudzki E (1976). Sensitivity to epoxy resins and triethylenetetramine. Contact Dermatitis.

[13] Arbogast JW, Fendler EJ, Hammond BS (2004). Effectiveness of a hand care regimen with moisturizer in manufacturing facilities where workers are prone to occupational irritant dermatitis. Dermatitis.

[14] Saary J, Qureshi R, Palda V (2005). A systematic review of contact dermatitis treatment and prevention. J Am Acad Dermatol.

[15] Williams C, Wilkinson SM, McShane P (2010). A double-blind, randomized study to assess the effectiveness of different moisturizers in preventing dermatitis induced by hand washing to simulate health care use. Br J Dermatol.

[16] Winker R, Salameh B, Stolkovich S (2009). Effectiveness of skin protection creams in the prevention of occupational dermatitis: results of a randomized, controlled trial. Int Arch Occup Environ Health.

[17] Williams JDL, Lee AYL, Matheson MC (2008). Occupational contact urticaria: Australian data. Br J Dermatol.

[18] Cox N, English J (2011). British Association of Dermatologists' Management Guidelines.

[19] Health and Safety Executive http://www.hse.gov.uk/riddor.

[20] van Wendel de Joode JB, Vermeulen R, Heederik D (2007). Evaluation of two self-administered questionnaires to ascertain dermatitis among metal workers and its relation with exposure to metal working fluids. Contact Dermatitis.

[21] Flyvholm MA, Mygind K, Sell L (2005). A randomised controlled intervention study on prevention of work related skin problems among gut cleaners in swine slaughterhouses. Occup Environ Med.

[22] Sell L, Flyvholm MA, Lindhard G, Mygind K (2005). Implementation of an occupational skin disease prevention programme in Danish cheese dairies. Contact Dermatitis.

[23] van der Walle HB (1994). Dermatitis in hairdressers (II), Management and prevention. Contact Dermatitis.

[24] Fischer T, Bjarnason B (1996). Sensitising and irritant properties of three environmental classes of diesel oil and their indicator dyes. Contact Dermatitis.

[25] Goh CL, Ho SF (1993). Contact dermatitis from dielectric fluids in electrodischarge machining. Contact Dermatitis.

[26] Cahill J, Keegel T, Dharmage S (2005). Prognosis of contact dermatitis in epoxy resin workers. Contact Dermatitis.

[27] Goh CL (1985). Occupational dermatitis from soldering flux among workers in the electronics industry. Contact Dermatitis.

[28] Rystedt I (1985). Atopic background in patients with occupational hand dermatitis. Contact Dermatitis.

[29] Stingeni L, Lapomarda V, Lisi P (1995). Occupational hand dermatitis in hospital environments. Contact Dermatitis.

[30] Dooms-Goossens A, Ceuterick A, Vanmaele N, Degreef H (1980). Follow-up study of patients with contact dermatitis caused by chromates, nickel, and cobalt. Dermatologica.

[31] Shah M, Lewis FM, Gawkrodger DJ (1996). Prognosis of occupational hand dermatitis in metalworkers. Contact Dermatitis.

[32] Adisesh A, Meyer JD, Cherry NM (2002). Prognosis and work absence due to occupational contact dermatitis. Outcome of cases reported to EPIDERM. Contact Dermatitis.

[33] Baack BR, Holguin TA, Holmes HS (1996). Use of a semi-permeable glove during treatment of hand dermatitis. Cutis.

[34] Kiec-Swierczynska M (1996). Occupational allergic contact dermatitis in Lodz: 1990–1994. Occup Med (Lond).

[35] Nethercott JR, Lawrence MJ, Roy AM, Gibson BL (1984). Airborne contact urticaria due to sodium benzoate in a pharmaceutical manufacturing plant. J Occup Med.

[36] Pedersen NB, Chevallier MA, Senning A (1982). Secondary acrylamides in Nyloprint printing plate as a source of contact dermatitis. Contact Dermatitis.

[37] Holness DL, Nethercott JR (1991). Is a worker's understanding of their diagnosis an important determinant of outcome in occupational contact dermatitis?. Contact Dermatitis.

[38] Kalimo K, Kautiainen H, Niskanen T, Niemi L (1999). ‘Eczema school’ to improve compliance in an occupational dermatology clinic. Contact Dermatitis.

[39] Agner T, Andersen K, Brandao F (2008). Hand eczema severity and quality of life: a cross-sectional, multicentre study of hand eczema patients. Contact Dermatitis.

[40] Skoet R, Zachariae R, Agner T (2003). Contact dermatitis and quality of life: a structured review of the literature. Br J Dermatol.

